# Hepatitis C Virus Infection Causes Iron Deficiency in Huh7.5.1 Cells

**DOI:** 10.1371/journal.pone.0083307

**Published:** 2013-12-13

**Authors:** Carine Fillebeen, Kostas Pantopoulos

**Affiliations:** 1 Lady Davis Institute for Medical Research, Jewish General Hospital, Montreal, Quebec, Canada; 2 Department of Medicine, McGill University, Montreal, Quebec, Canada; Karolinska Institutet, Sweden

## Abstract

Patients with chronic hepatitis C virus (HCV) infection frequently develop systemic iron overload, which exacerbates morbidity. Nevertheless, iron inhibits HCV replication in cell culture models and thereby exerts antiviral activity. We hypothesized that the cellular iron status is crucial for the establishment of HCV infection. We show that HCV infection of permissive Huh7.5.1 hepatoma cells promotes an iron deficient phenotype. Thus, HCV leads to increased iron regulatory protein (IRP) activity, accumulation of IRP2 and suppression of transferrin receptor 1 (TfR1) and divalent metal transporter 1 (DMT1) in the host. These data suggest that HCV regulates cellular iron levels to bypass iron-mediated inhibition in viral replication.

## Introduction

HCV infection continues to pose a global health concern, with an estimated prevalence of 2.2–3% worldwide [1]. Progression to chronic hepatitis C (CHC) predisposes to liver fibrosis, cirrhosis and hepatocellular cancer. CHC patients often present with elevated serum iron indices and hepatic iron overload, which is in its own right a risk factor for liver disease [2]. Misregulation of iron homeostasis in CHC is caused by many factors, including necroinflammation and suppression of the iron regulatory hormone hepcidin [2]. Hepcidin insufficiency promotes dietary iron absorption and efflux of iron from reticuloendothelial macrophages to the bloodstream, while excess iron eventually accumulates within hepatocytes [3]. Paradoxically, while iron aggravates HCV toxicity, it may also exhibit antiviral activity [4].

We previously showed that iron impairs the enzymatic activity of the viral RNA polymerase NS5B [5] and thereby inhibits replication of subgenomic [5] and infectious HCV [6]. Furthermore, we reported that subgenomic HCV replicons reduce iron levels in host cells [7]. Here, we address the effects of infectious HCV on iron metabolism in permissive Huh7.5.1 hepatoma cells. We demonstrate that these cells express inappropriately low hepcidin mRNA levels and develop an iron deficient phenotype in response to HCV infection.

## Materials and Methods

### Cell culture

Huh7.5.1 cells were cultured in Dulbecco's modified Eagle's medium supplemented with 10% heat inactivated fetal bovine serum, 100 nM non-essential amino acids, 100 U/ml penicillin and 100 µg/ml streptomycin.

### Infection of Huh7.5.1 cells with HCV

In vitro transcribed HCV RNA (derived from clone JFH-1) was transfected into Huh7.5.1 cells by electroporation [6]. After 14 days, culture media containing viral particles were cleared by low speed centrifugation, filtered and used for inoculation of naïve Huh7.5.1 cells. The infected cells were washed after 24 hours and incubated with fresh media for 1–4 days.

### Western blotting

The expression of viral and cellular proteins was analyzed by Western blotting [6], [7]. Immunoreactive bands were quantified by densitometry and values were normalized to those of control β-actin.

### Quantitative real-time RT-PCR

HCV RNA and the expression of cellular mRNAs were quantified by real time PCR, following reverse transcription [6], [7]. Values were normalized to those of ribosomal protein S18 (RPS18) RNA. Primer sequences are shown in Table 1.

**Table 1:** List of primers used for qPCR.

Gene	GenBank accession No	Forward primer sequence	Reverse primer sequence
RPS18	NM 022551	tgtggtgttgaggaaagcag	aagtgacgcagccctctatg
HCV		ctgtcttcacgcagaaagcg	cactcgaccgcgccctatca
Hamp (hepcidin)	NM 021175	atggcactgagctcccagat	actttgatcgatgacagcag
TfR1	NM 003234	gcaagtagatggcgataacag	gacgatcacagcaatagtccc
DMT1+IRE	NM 001174125	gtggtcagcgtggcttatct	cacactggctctgatggcta

### Electrophoretic mobility shift assay (EMSA)

IRE-binding activities of IRP1 and IRP2 were analyzed by EMSA with a ^32^P-labeled IRE probe [8]. IRE/IRP1 and IRE/IRP2 complexes, which co-migrate in the gels under the specific running conditions if IRP1 and IRP2 are of human origin, were visualized by autoradiography. IRE-binding activities were quantified by phosphorimaging and normalized to values obtained in the presence of 2% mercaptoethanol, which activates latent IRP1 [8].

### Statistical Analysis

Data are shown as means ±SD. Statistical analysis was performed by the unpaired Student's t-test with the Prism GraphPad Software (version 5.0 d).

## Results and Discussion

**Figure 1:**
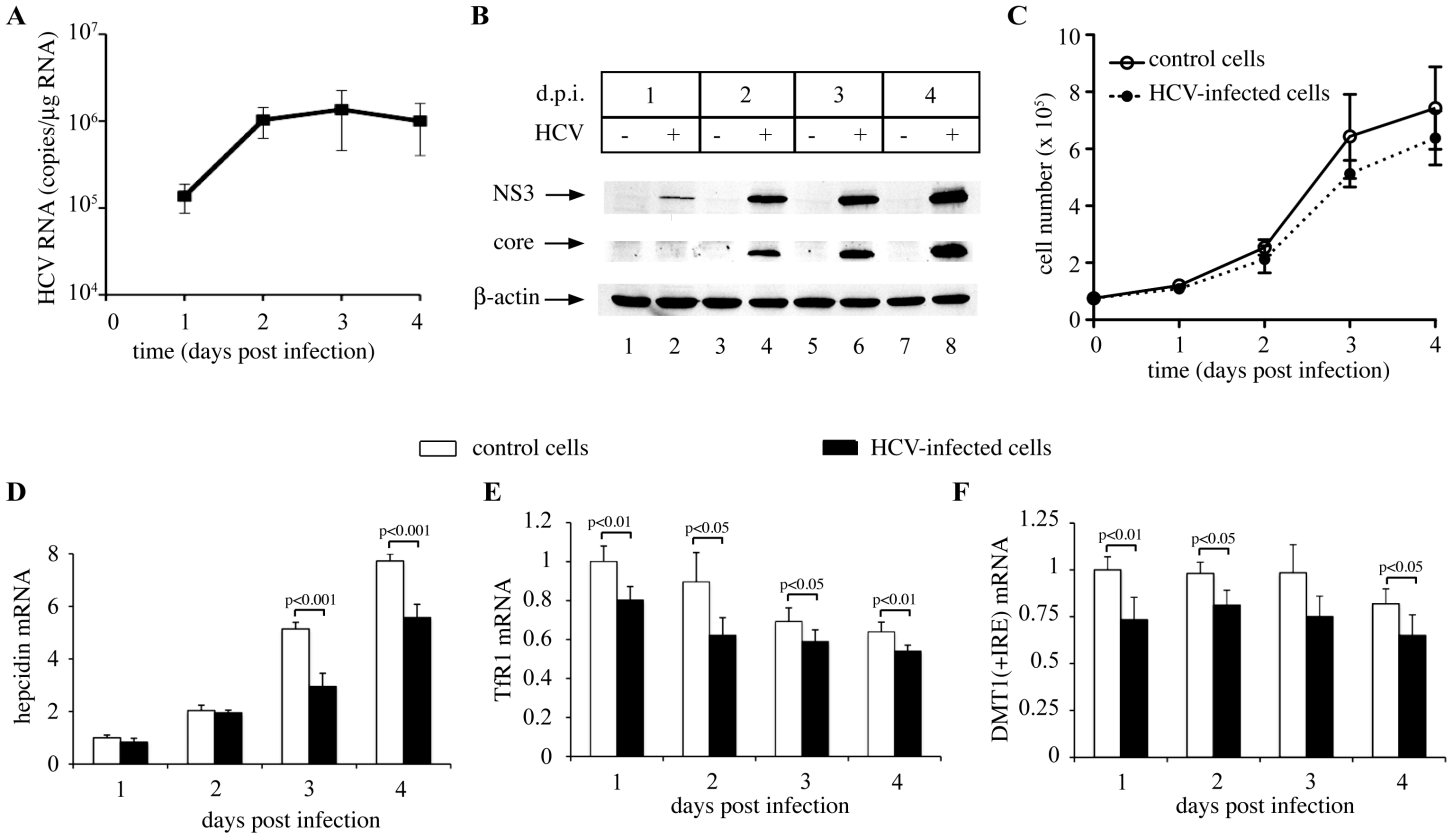
HCV infection suppresses the expression of hepcidin, TfR1 and DMT1 mRNAs. Naïve Huh7.5.1 cells were inoculated with media containing HCV particles or control media for 1–4 days post infection (d.p.i.). (A) The expression of HCV RNA was quantified by qPCR. (B) The expression of viral NS3 and core proteins and of cellular β-actin was analyzed by Western blotting. (C) The growth rate of uninfected control and HCV-infected cells was monitored by counting viable cells with the trypan blue exclusion method (n = 4 experiments). (D–E) The levels of hepcidin, TfR1 and DMT1(+IRE) mRNAs were analyzed by qPCR. After normalization with RPS18 values, mRNAs in HCV-infected cells were expressed relative to control cells at day 1. The graphs represented three independent experiments (means ±SD).

Infection of naïve Huh7.5.1 cells with HCV was accomplished by their inoculation with culture supernatant from HCV RNA-transfected Huh7.5.1 cells. The efficacy of this procedure is demonstrated by the robust expression of HCV RNA (Fig. 1A) and the viral NS3 and core proteins (Fig. 1B). Uninfected control and HCV-infected cells were supplemented with fresh media and allowed to grow for 1–4 days. Growth of HCV-infected cells was slower after day 2 (Fig. 1C). Hepcidin mRNA levels were minimal on day 1, but gradually increased afterwards (Fig. 1D). This was likely due to time-dependent inactivation of growth factors present in the culture media, since these molecules inhibit hepcidin transcription [9]. Importantly, the recovery of hepcidin mRNA was significantly blunted in HCV-infected cells (p<0.005 on days 3 and 4), suggesting that HCV infection inhibits hepcidin mRNA expression. This result is consistent with the reduced serum hepcidin levels in CHC patients [10], the transcriptional inactivation of hepcidin in full-length HCV Huh7.5 replicon cells [11], and the impairment of hepcidin mRNA expression in primary human hepatocytes following in vitro infection with HCV [12].

Both uninfected control and HCV-infected cells exhibited a time-dependent decline in transferrin receptor 1 (TfR1) mRNA expression (Fig. 1E). This response probably reflected the gradual increases in cell density, which negatively regulates basal TfR1 transcription [13]. TfR1 mRNA levels were significantly lower in HCV-infected cells compared to uninfected controls throughout the experiment, in spite of their decreased growth rate. This is in line with the reduced TfR1 mRNA expression previously documented in subgenomic HCV replicon cells [7], and recently in HCV-infected Huh7 cells [14]. Expression of an iron-regulated isoform of divalent metal 1 transporter (DMT1) mRNA was likewise reduced in HCV-infected cells (Fig. 1F).

**Figure 2:**
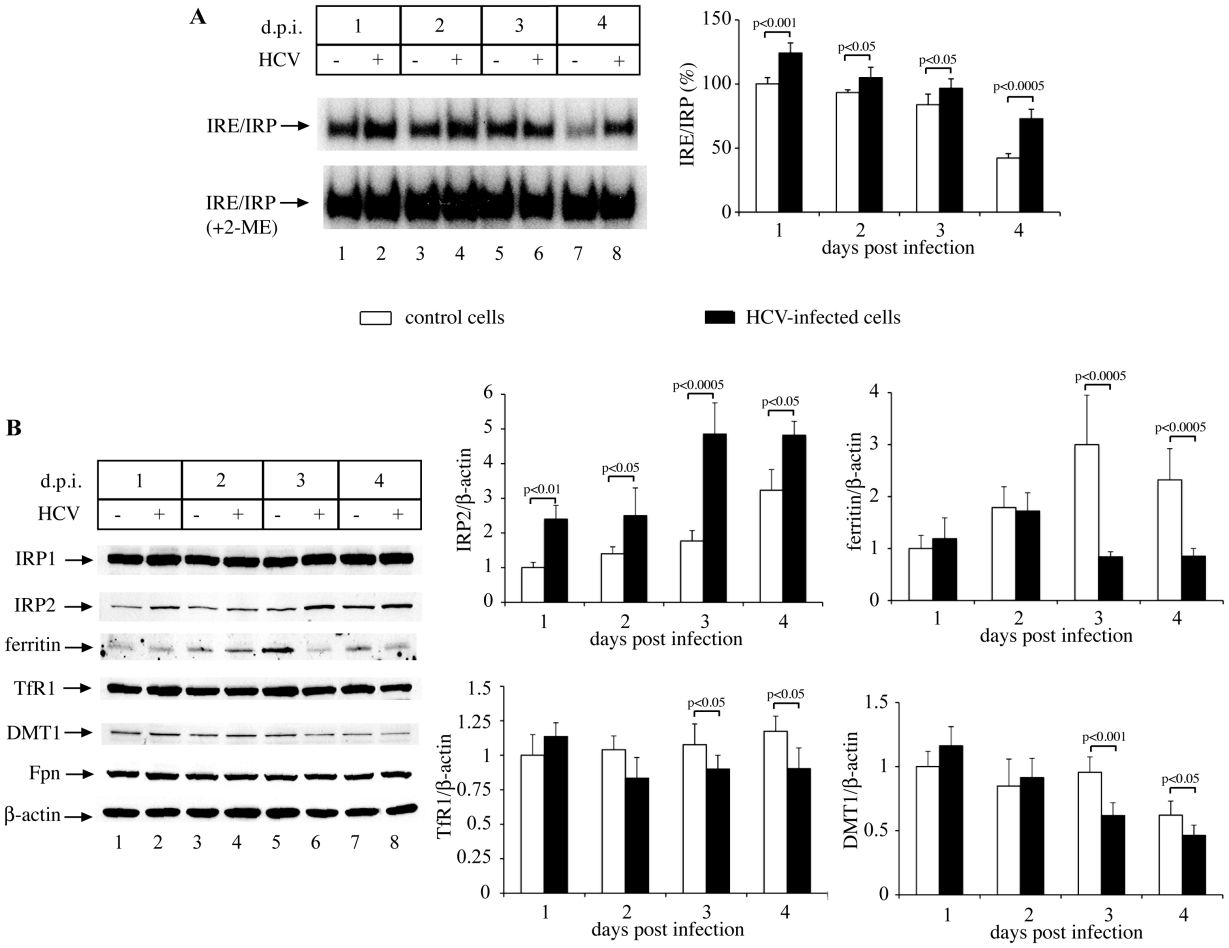
HCV infection activates IRPs and inhibits the expression of iron uptake molecules. Huh7.5.1 cells were inoculated with media containing HCV particles for 1–4 days post infection (d.p.i.). (A) Cell lysates were analyzed by EMSA with a ^32^P-labeled IRE probe in the absence (top) or presence (bottom) of 2% 2-mercaptoethanol (2-ME). Data from three independent experiments were quantified by densitometry. The graph depicts percentages of IRE/IRP band intensities, normalized to the respective 2-ME values (means ±SD). (B) The expression of IRP1, IRP2, TfR1, ferritin, Fpn, DMT1, and β-actin was analyzed by Western blotting. Data from three independent experiments were quantified by densitometry; relative protein band intensities (means ±SD) are plotted on the right, following normalization with the respective β-actin values.

Both TfR1 and DMT1 are involved in cellular iron uptake [15], while TfR1 appears to further operate as an HCV entry factor [14]. The downregulation of TfR1 and DMT1 mRNAs in HCV-infected cells may lead to iron deficiency. This state is sensed by iron regulatory proteins, IRP1 and IRP2, which bind to mRNAs containing iron responsive elements (IREs) and thereby control their translation or stability [15]. IRE-binding activity was consistently and significantly elevated in HCV-infected cells (Fig. 2A), which indicates an iron-deficient phenotype. Notably, IRE-binding activity tended to decrease in a time-dependent manner, possibly as a result of increasing cell density [16].

In iron-deficient cells, IRP1 is activated for IRE-binding upon removal of its iron-sulfur cluster, while IRP2 activation is due to its stabilization against degradation [15]. IRP1 expression was similar in control and HCV-infected cells (Fig. 2B, top), in agreement with the regulation of its IRE-binding activity via an iron-sulfur cluster switch. By contrast, IRP2 content was significantly elevated in HCV-infected cells (Fig. 2B, second panel). Together with the increased IRE-binding activity, the induction of IRP2 provides strong evidence that HCV-infected cells are iron-deficient. This interpretation is also supported by the concomitant inhibition in the expression of ferritin (Fig. 2B, third panel), an iron storage protein that is negatively regulated by IRPs [15].

HCV-dependent iron deficiency could be caused by the reduced expression of the iron uptake proteins TfR1 and DMT1 (Fig. 2B, fourth and fifth panels, respectively), which largely reflects their mRNA content (Fig. 1). The levels of the iron exporter ferroportin (Fpn) were not significantly affected (Fig. 2B, sixth panel; quantification is shown in Fig. S1), suggesting that HCV infection does not directly interfere with iron efflux. As both TfR1 and DMT1 are positively regulated by IRPs, their reduced expression in HCV-infected cells appears to be IRP-independent.

In conclusion, our findings provide evidence that HCV infection modulates iron metabolism and promotes iron deficiency in host hepatic cells. Similar responses were elicited by subgenomic HCV replicons; nevertheless, in this setting, iron deficiency was also associated with Fpn induction [7]. Conceivably, Fpn expression may be regulated by HCV structural proteins. Considering that iron antagonizes the establishment of HCV infection by inhibiting viral replication [5], [6], the capacity of HCV to promote iron deficiency in host cells may represent an adaptive strategy to bypass the antiviral activity of iron. The delayed suppression of TfR1 by HCV is not expected to compromise viral entry, since TfR1 appears to be essential for internalization of HCV particles only at early stages of infection [14].

## Supporting Information

Figure S1 **HCV infection does not significantly alter ferroportin expression in host Huh7.5.1 cells.** Huh7.5.1 cells were inoculated with media containing HCV particles for 1–4 days post infection. The expression of ferroportin was analyzed by Western blotting. Data from three independent experiments, including that shown in Fig. 2B, were quantified by densitometry. The graph depicts relative ferroportin band intensities (means ±SD) normalized to β-actin.
(TIF)

## References

[1] Lavanchy D (2009) The global burden of hepatitis C. Liver Int 29 Suppl 174–81. 10.1111/j.1478-3231.2008.01934.x 19207969

[2] Sebastiani G, Pantopoulos K (2011) Disorders associated with systemic or local iron overload: from pathophysiology to clinical practice. Metallomics 3: 971–986. 21901209 10.1039/c1mt00082a

[3] Ganz T, Nemeth E (2012) Hepcidin and iron homeostasis. Biochim Biophys Acta 1823: 1434–1443. 22306005 10.1016/j.bbamcr.2012.01.014 PMC4048856

[4] Mueller S (2010) Increased iron in HCV infection: Collateral damage or antiviral defense? J Hepatol. 10.1016/j.jhep.2010.08.003 20850193

[5] Fillebeen C, Rivas-Estilla AM, Bisaillon M, Ponka P, Muckenthaler M, et al (2005) Iron inactivates the RNA polymerase NS5B and suppresses subgenomic replication of hepatitis C virus. J Biol Chem 280: 9049–9057. 15637067 10.1074/jbc.M412687200

[6] Fillebeen C, Pantopoulos K (2010) Iron inhibits replication of infectious hepatitis C virus in permissive Huh7.5.1 cells. J Hepatol 53: 995–999. 20813419 10.1016/j.jhep.2010.04.044

[7] Fillebeen C, Muckenthaler M, Andriopoulos B, Bisaillon M, Mounir Z, et al (2007) Expression of the subgenomic hepatitis C virus replicon alters iron homeostasis in Huh7 cells. J Hepatol 47: 12–22. 17399844 10.1016/j.jhep.2007.01.035

[8] Mueller S, Pantopoulos K (2002) Activation of iron regulatory protein-1 (IRP1) by oxidative stress. Methods Enzymol 348: 324–337. 11885287 10.1016/s0076-6879(02)48651-x

[9] Goodnough JB, Ramos E, Nemeth E, Ganz T (2012) Inhibition of hepcidin transcription by growth factors. Hepatology 56: 291–299. 22278715 10.1002/hep.25615 PMC3362690

[10] Girelli D, Pasino M, Goodnough JB, Nemeth E, Guido M, et al (2009) Reduced serum hepcidin levels in patients with chronic hepatitis C. J Hepatol 51: 845–852. 19729219 10.1016/j.jhep.2009.06.027 PMC2761995

[11] Miura K, Taura K, Kodama Y, Schnabl B, Brenner DA (2008) Hepatitis C virus-induced oxidative stress suppresses hepcidin expression through increased histone deacetylase activity. Hepatology 48: 1420–1429. 18671304 10.1002/hep.22486

[12] Liu H, Trinh TL, Dong H, Keith R, Nelson D, et al (2012) Iron regulator hepcidin exhibits antiviral activity against hepatitis C virus. PLoS One 7: e46631. 23110054 10.1371/journal.pone.0046631 PMC3478283

[13] Wang J, Chen G, Pantopoulos K (2005) Inhibition of transferrin receptor 1 transcription by a cell density response element. Biochem J 392: 383–388. 16092918 10.1042/BJ20050492 PMC1316274

[14] Martin DN, Uprichard SL (2013) Identification of transferrin receptor 1 as a hepatitis C virus entry factor. Proc Natl Acad Sci U S A 110: 10777–10782. 23754414 10.1073/pnas.1301764110 PMC3696786

[15] Wang J, Pantopoulos K (2011) Regulation of cellular iron metabolism. Biochem J 434: 365–381. 21348856 10.1042/BJ20101825 PMC3048577

[16] Popovic Z, Templeton DM (2012) Cell density-dependent shift in activity of iron regulatory protein 1 (IRP-1)/cytosolic (c-)aconitase. Metallomics 4: 693–699. 22544036 10.1039/c2mt20027a

