# Supplementary figures and images for: Hepatitis C Virus Infection Causes Iron Deficiency in Huh7.5.1 Cells
Source: PLoS One. 2013 Dec 13;8(12):e83307. doi: 10.1371/journal.pone.0083307 (PMC3862679; doi:10.1371/journal.pone.0083307)

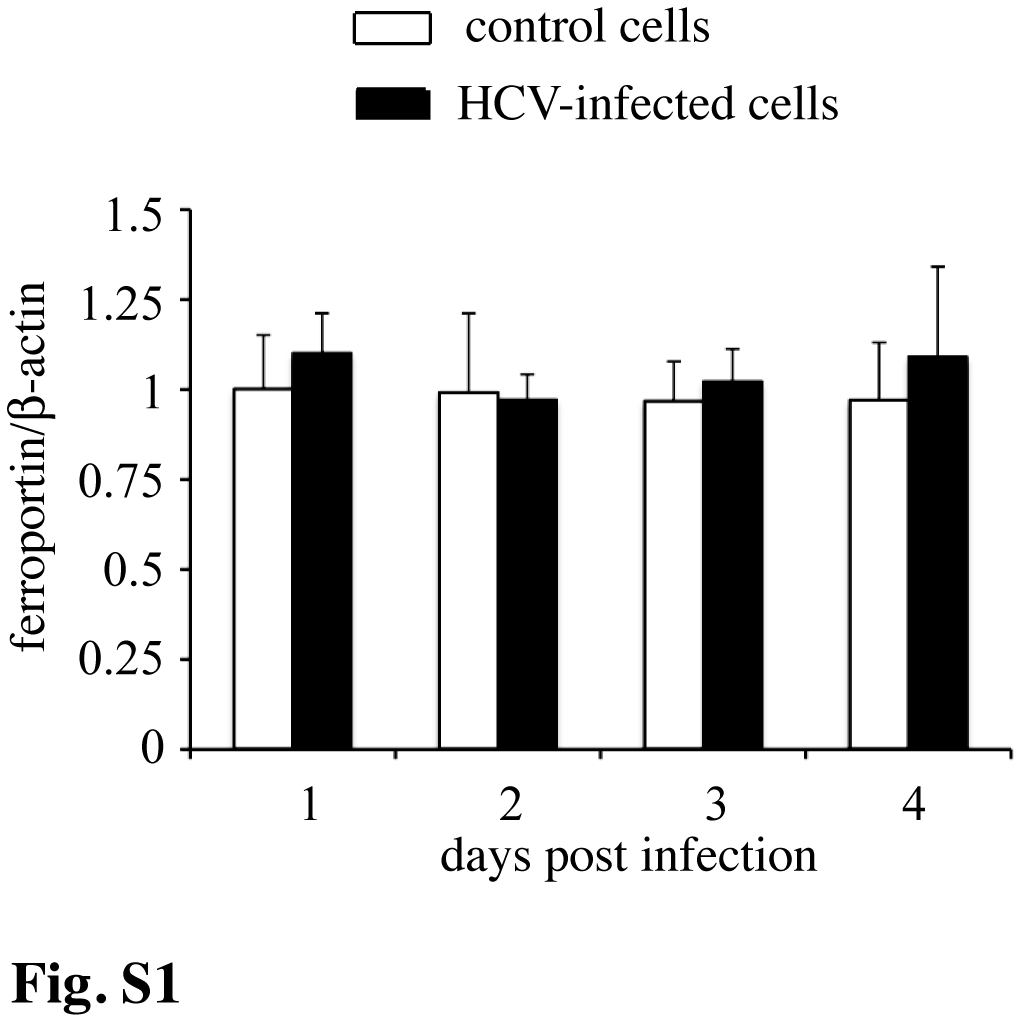

Supplement: Figure S1 — HCV infection does not significantly alter ferroportin expression in host Huh7.5.1 cells. Huh7.5.1 cells were inoculated with media containing HCV particles for 1–4 days post infection. The expression of ferroportin was analyzed by Western blotting. Data from three independent experiments, including that shown in Fig. 2B, were quantified by densitometry. The graph depicts relative ferroportin band intensities (means ±SD) normalized to β-actin. (TIF) [file pone.0083307.s001.tif]
